# Sodium-glucose co-transporter 2 inhibition as a mitochondrial therapy for atrial fibrillation in patients with diabetes?

**DOI:** 10.1186/s12933-019-0984-0

**Published:** 2020-01-07

**Authors:** Salva R. Yurista, Herman H. W. Silljé, Michiel Rienstra, Rudolf A. de Boer, B. Daan Westenbrink

**Affiliations:** Department of Cardiology, University of Groningen, University Medical Center Groningen, PO Box 30.001, 9700 RB Groningen, The Netherlands

**Keywords:** Mitochondria, Diabetes, Atrial fibrillation, Sodium-glucose co-transporter-2 inhibitors

## Abstract

While patients with type 2 diabetes mellitus (T2DM) are at increased risk to develop atrial fibrillation (AF), the mechanistic link between T2DM and AF-susceptibility remains unclear. Common co-morbidities of T2DM, particularly hypertension, may drive AF in the setting of T2DM. But direct mechanisms may also explain this relation, at least in part. In this regard, recent evidence suggests that mitochondrial dysfunction drives structural, electrical and contractile remodelling of atrial tissue in patients T2DM. Mitochondrial dysfunction may therefore be the mechanistic link between T2DM and AF and could also serve as a therapeutic target. An elegant series of experiments published in *Cardiovascular Diabetology* provide compelling new evidence to support this hypothesis. Using a model of high fat diet (HFD) and low-dose streptozotocin (STZ) injection, Shao et al. provide data that demonstrate a direct association between mitochondrial dysfunction and the susceptibility to develop AF. But the authors also demonstrated that the sodium-glucose co-transporter 2 inhibitors (SGLT2i) empagliflozin has the capacity to restore mitochondrial function, ameliorate electrical and structural remodelling and prevent AF. These findings provide a new horizon in which mitochondrial targeted therapies could serve as a new class of antiarrhythmic drugs.

## Introduction

Type 2 diabetes mellitus (T2DM) is a major cardiovascular (CV) risk factor, and its global prevalence is predicted to increase from 425 million to 600 million by the year 2045 [1]. The projected number of individuals with atrial fibrillation (AF) in the European Union could reach 14–17 million by 2030 [2]. T2DM and AF have both emerged as cardiometabolic epidemics [1, 2]. Patients with T2D are at a 40% increased risk to develop new-onset AF [3–5] and the risk of new-onset AF increased gradually with advancing diabetic stage [6]. Furthermore, patients with T2D and AF are also at increased risk to for complications of AF such as stroke and systemic embolisms and hospitalisations for heart failure (HF) [7–9]. In addition, the evidence has suggested that these patients may actually benefit from the use of non-vitamin K oral anticoagulants (NOACs) given the demonstrated efficacy and improved safety profile as compared to warfarin [10]. This improved safety profile was also confirmed in ARISTOTLE trial [11].

The mechanism responsible for the high incidence and increased severity of AF in patients with T2DM is the subject of intense speculation but remains largely enigmatic. Patients with AF and T2DM share common comorbidities such as hypertension, atherosclerosis and obesity [12]. Targeted therapy of risk factors has been shown to improve AF outcomes [13]. An observational cohort study from Korean National Health Insurance Service database suggests avoiding body weight fluctuation, regardless weight gain or weight loss, is important to prevent AF development and to decrease the risk [14, 15].

Interestingly, an experimental study by Chen et al. showed that insulin resistance promotes interstitial fibrosis and alters calcium handling that induce arrhythmogenesis in the atria [16]. Morphological and functional comparisons of atrial tissue from patients with or without diabetes have revealed that fibrosis was more elevated in diabetic atria [17]. Furthermore, atria from patients with T2DM and AF consistently display evidence for increased oxidative stress, suggesting that the oxidative stress and/or underlying mechanisms may represent a T2DM-specific therapeutic target for AF [18, 19].

The myocardium requires tremendous amounts of energy in the form of adenosine triphosphate (ATP) to sustain its continuous mechanical work [20]. The majority of this energy is generated through oxidative phosphorylation in mitochondria, which comprise about 30% of the myocardial volume. Mitochondrial energy provision is not only essential for contraction and relaxation, but calcium handling by the sarcoplasmic reticulum and ion channel homeostasis are also critically dependent on ATP availability. In addition, mitochondria also important myocellular storage compartments and alterations in mitochondrial calcium handling contribute to arrhythmogenesis, pathological cardiac remodelling, and apoptosis. Mitochondria are also the main cardiac source of reactive oxygen species (ROS), which originate from the electron transport chain during oxidative phosphorylation. Under physiological conditions ROS-induced myocardial damage is minimized through tight control of the mitochondrial redox balance and an efficient and dynamic mitochondrial quality control program. Mitochondrial quality control/mitochondrial dynamics ensure the fitness of the mitochondrial population through continuous quality checks, the elimination of dysfunctional mitochondrial and promoting growth of new organelles [21].

In many patients with heart disease these protective mechanisms fall short, resulting in increases in mitochondrial ROS, reductions in myocardial ATP and the accumulation dysfunctional mitochondria. While mitochondrial dysfunction has been recognised as a therapeutic target in other heart diseases such as heart failure, the role of mitochondrial dysfunction in arrhythmogenesis is not well described. In an elegant study published in *Cardiovascular Diabetology*, Shao et al. confirm and extend upon previous evidence for a mechanistic link between T2DM, mitochondrial dysfunction and AF [22]. In addition, and rendering translational importance, the authors demonstrate that the sodium-glucose co-transporter 2 inhibitors (SGLT2i) empagliflozin can reverse mitochondrial dysfunction and ameliorate the susceptibility to develop AF in rats with T2DM. Together, these findings indicate that mitochondrial dysfunction is a potentially treatable cause of AF, for which therapeutic interventions are already available. In the current commentary we will summarize contemporary evidence for the role of mitochondria in arrhythmogenesis in patients with AF and also discuss the therapeutic perspectives provided by the study by Shao et al. [22].

## Mitochondrial dysfunction in T2DM and AF

Mitochondrial dysfunction has been described in many organs of patients with T2DM, including the atria [23]. For instance, mitochondria isolated from the atria of patients with diabetes display reduced mitochondrial respiration and increased oxidative stress, when compared to subjects without diabetes [24]. The mitochondrial architecture and the assembly of the electron transport chain are also altered in patients with T2DM and these ultrastructural changes appear to be even more pronounced in the presence of AF, suggesting a reciprocal relation [25].

Indeed, abnormal mitochondrial structure and function have been reported in animal model of AF [26], Moreover, the atria of non-diabetic patients with AF already display enhanced mitochondrial DNA damage [27, 28], and reduced respiratory capacity [27, 29]. Mitochondrial dynamics are also altered in patients with AF, characterized by a reduction in mitochondrial biogenesis [30]. Specifically, Jeganathan et al. observed that the main regulator of mitochondrial biogenesis peroxisome proliferator-activated receptor gamma coactivator 1-alpha (PGC-1α) is downregulated in atrial tissue from patients with post-operative AF [30]. Furthermore, molecular markers for mitochondrial volume are also reduced in the atrial tissue from patients with AF [31]. It remains uncertain whether the observed mitochondrial dysfunction is a cause or a consequence of AF.

## How does mitochondrial dysfunction lead to AF?

As described above, dysfunctional mitochondria are less able to generate ATP and produce more ROS. Excessive ROS production can disturb cellular electrical activity in two ways. First, ROS has pro-arrhythmic effects by modulating redox-sensitive regulatory domains of multiple proteins involves in excitation contraction coupling, including sarcoendoplasmic reticulum (SR) calcium transport ATPase (SERCA), Na^+^ channels, K^+^ channels, L-type Ca^2+^ channels (LCCs), ryanodine receptors (RyRs), Na^+^/Ca^2+^ exchanger (NCX) [32–36]. In addition, ROS can also directly activate signalling such as Ca^2+^/calmodulin dependent kinase II (CaMKII). CaMKII is a multifunctional protein that serves as a nodal regulator of many cellular responses, including excitation–contraction coupling, excitation–metabolism coupling and excitation–transcription coupling [37–40]. CaMKII can be activated by multiple stimuli, including but not restricted to sustained increases in mitochondrial ROS and hyperglycaemia [37, 41]. The combination of hyperglycaemia and increased ROS which occurs in diabetic atria sets the ideal stage for robust and sustained CaMKII activation, which has been identified as a major driver of arrhythmogenicity in diabetic hearts, and may at least partially explain the high incidence of AF in patients with diabetes [41].

Bioenergetic deficiencies caused by mitochondrial dysfunction may also result in impediments in ion channels homeostasis. [42]. Reductions in ATP levels can lead to the activations of sarcoplasmic ATP-sensitive potassium (K_ATP_) channels, causing shortening of action potential duration (APD) and reduction of action potential amplitude (APA) [43]. Furthermore, reduced mitochondrial ATP production suppresses the activity of SERCA and Na^+^/K^+^ ATPase, which will alter calcium (Ca^2+^) handling [44, 45] and increase the susceptibility to develop AF [46].

**Fig. 1:**
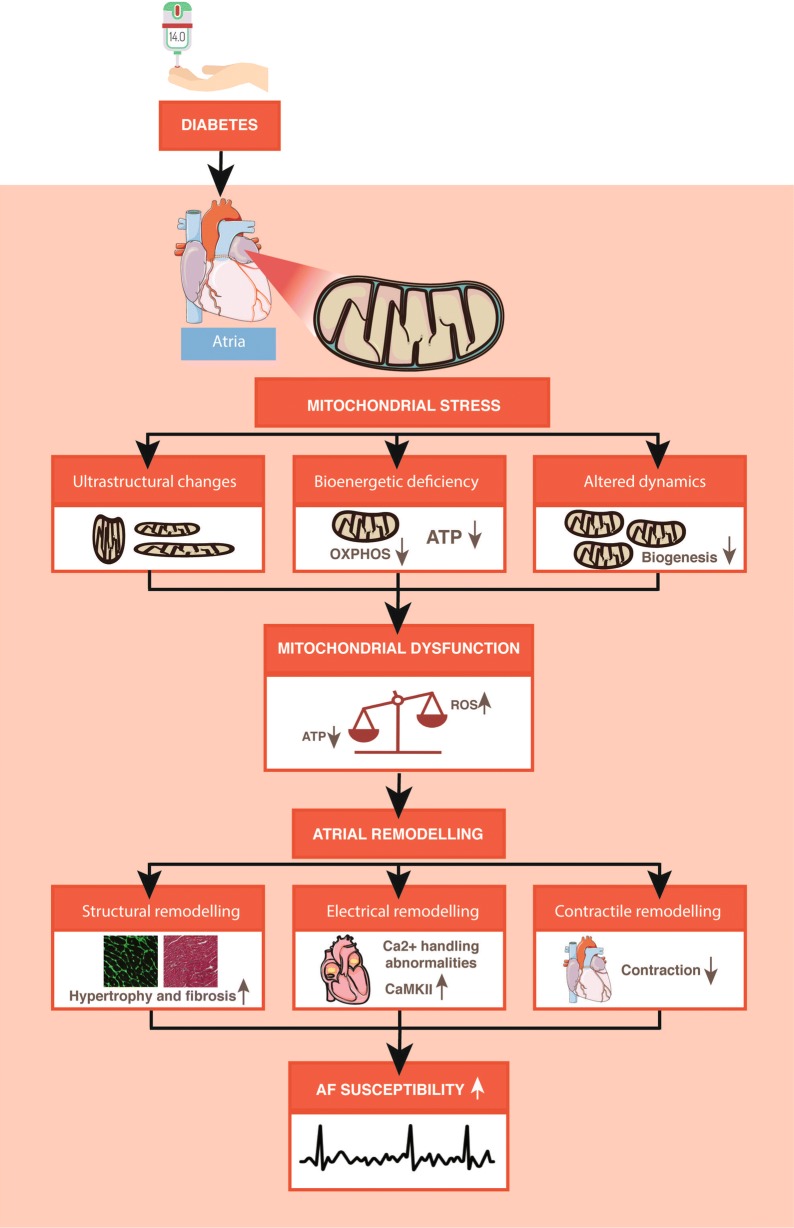
Contribution of diabetes to pathophysiology of atrial fibrillation. *OXPHOS* oxidative phosphorylation, *ATP* adenosine triphosphate, *ROS* reactive oxygen species, *CaMKII* Ca^2+^/calmodulin dependent kinase II, *AF* atrial fibrillation. Part of illustration elements courtesy of Servier Medical Art

Finally, oxidative stress and bioenergetic deficiencies can also promote cardiomyocyte hypertrophy and interstitial fibrosis, two central drivers of atrial remodelling that promote AF [47]. As described above, atrial remodelling is a hallmark of AF and the degree of atrial remodelling is more pronounced in individuals with diabetes [48]. In summary, mitochondrial dysfunction in atria from diabetic subject can promote AF through multiple mechanisms summarized in Fig. 1. These findings suggest that targeting mitochondria could represent a feasible therapeutic strategy to reduce the burden of AF in diabetic patients.

SGLT2i are designed to reduce hyperglycaemia [49] but have been shown to improve mitochondrial function in ventricular myocardium of diabetic and non-diabetic animal models of heart failure [50, 51]. Dr. Shao et al. tested the hypothesis that these drugs may also preserve mitochondrial function and reduce atrial remodelling in diabetic atria [22]. For this purpose, they employed a combination of high fat diet (HFD) and low-dose streptozotocin (STZ) injection to induce T2DM in male rats. HFD and low-dose of STZ model has been used as a reasonable animal model of T2DM. Similar to pathophysiology in human, this model demonstrates the progression from insulin resistance to hypoinsulinemia and hyperglycaemia [52].

Animals with non-fasting blood glucose levels above 16.7 mmol/l measured 1 week after STZ injection were considered diabetic. Diabetic rats were then randomized to intragastric administration of empagliflozin (10 or 30 mg/kg/day) or vehicle for the duration of 8 weeks. Rats on a normal diet that did not receive HFD or STZ served as controls. After 8 weeks, cardiac structure and function were measured by echocardiography and a Millar conductance catheter. After sacrifice, atrial tissue was harvested to study histological and molecular indices of atrial remodelling and mitochondrial dynamics. In addition, mitochondria were isolated and their respiratory capacity and membrane potential was probed with the Oroboros system. In separate series of experiments, the hearts were excised and retrogradely perfused using a Langendorff setup to test AF-susceptibility with a well-established burst pacing protocol.

As expected, empagliflozin lowered blood glucose levels and reduced body weight. Moreover, treatment with high dose empagliflozin prevented LA enlargement and reduced cardiomyocyte hypertrophy and interstitial fibrosis. The susceptibility to AF was also normalized to control levels. Empagliflozin reduced oxidative stress as evidenced by increased superoxide dismutase (SOD) activity and reduced malondialdehyde (MDA) concentrations. Furthermore, the reductions in mitochondrial respiration and mitochondrial membrane potential which occurred in diabetic animals were restored to control levels by empagliflozin. Finally, the recovery of mitochondrial function by empagliflozin were accompanied by similar improvements in mitochondrial dynamics.

The study by Shao et al. [22] is worth noticing for several reasons.

First, most studies with SGLT2i have focussed on ventricular myocardium. The current study is the first to show that SGLT2i prevent electrical and structural remodelling of atria and reduces the propensity to develop AF. It was recently shown that SGLT2i can improve outcome in heart failure patients with or without diabetes [53]. Mitochondrial dysfunction and atrial remodelling are relatively independent of the presence of diabetes and similar mito-protective effects have been observed in non-diabetic models. The beneficial effects of SGLT2i could therefore also translate into similar generic benefits patients with AF. Nevertheless, it is also possible that the benefits on the atria occur via changes in plasma metabolites or other indirect effects. Thus, further research is required to confirm this hypothesis.

Second, while several studies have provided suggestive evidence that empagliflozin improves myocardial function, the authors are the first to convincingly show that SGLT2i improve mitochondrial respiration at the organelle level. In addition, the authors are the first to demonstrate that these mito-protective effects also occur in the atrium. In addition, the authors provide evidence that the favourable mitochondrial effects of SGLT2i have the propensity to reduce the burden of AF. Of note, a meta-analysis of 35 studies that included 34,987 T2DM patients showed no difference in AF occurrence between SGLT2i and placebo [54].

## Summary and conclusions

**Fig. 2:**
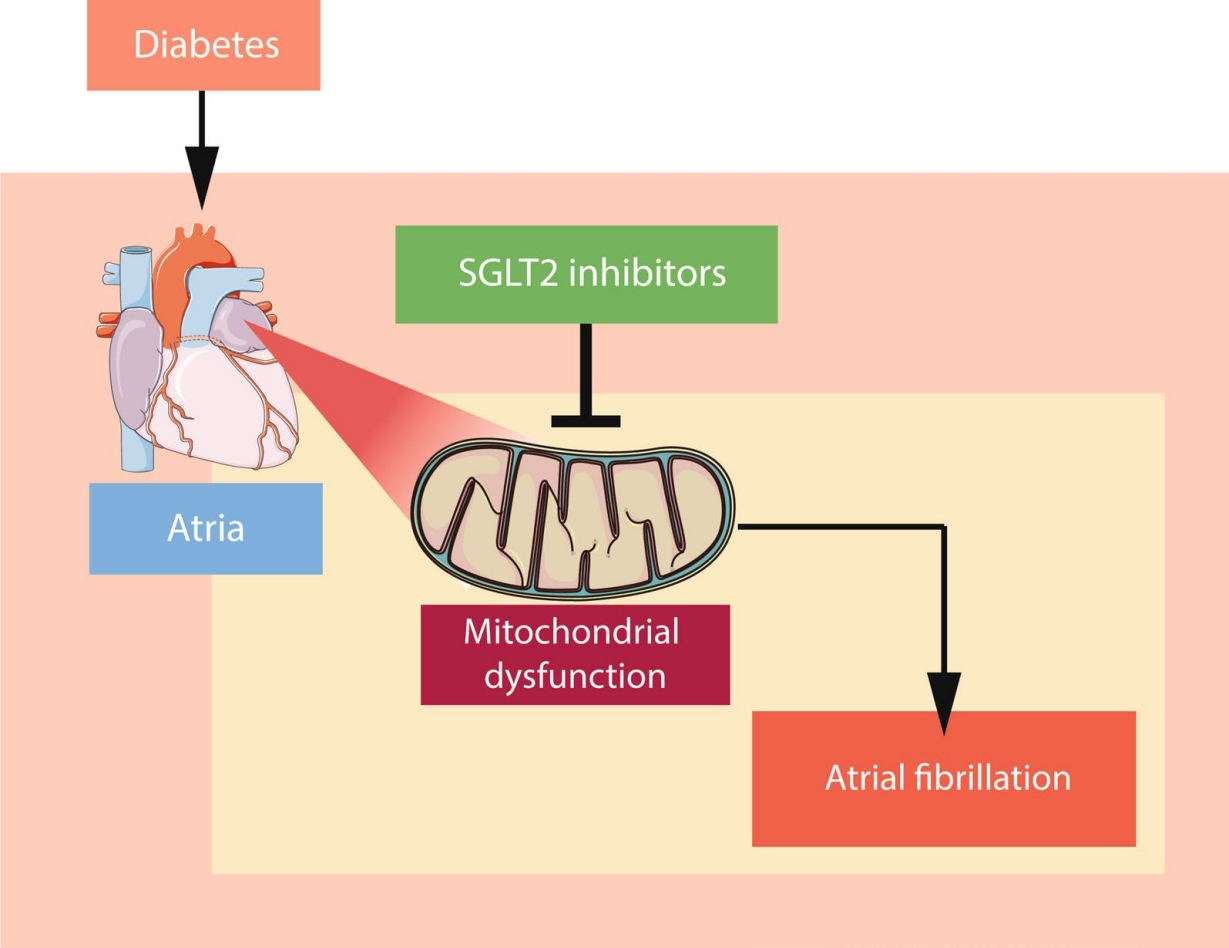
Proposed mechanisms for a SGLT2 inhibitors-induced antiarrhythmic effect in diabetes. *SGLT2* sodium-glucose co-transporter 2. Part of illustration elements courtesy of Servier Medical Art

In summary, the present study has extended our knowledge on the effects of SGLT2i and empagliflozin on atrial electrical and structural remodelling in diabetic setting. It provides compelling evidence that mitochondrial dysfunction could serve as a promising therapeutic target in AF, at least in diabetic patients. A proposed mechanism illustrating how SGLT2i could prevent AF in T2DM is shown in Fig. 2. Indeed, further mechanistic studies in both human and animals to better understand the benefits and potential application are warranted. Post-hoc analyses of ongoing and upcoming trials may also help to better define the scope of clinical effects of SGLT2i in patients with prevalent AF and to evaluate their effects on new onset AF. The current analysis provides a first step that may lead to mitochondrial targeted therapy for the treatments of AF in patients with diabetes?

## Data Availability

Not applicable.

## Abbreviations

T2DM: type 2 diabetes mellitus
AF: atrial fibrillation
HF: heart failure
ATP: adenosine triphosphate
ROS: reactive oxygen species
SGLT2i: sodium-glucose co-transporter 2 inhibitors
PGC-1α: peroxisome proliferator-activated receptor gamma coactivator 1-alpha
SERCA: sarcoendoplasmic reticulum (SR) calcium transport ATPase
LCCs: L-type Ca^2+^ channels
RyRs: ryanodine receptors
NCX: Na^+^/Ca^2+^ exchanger
CaMKII: Ca^2+^/calmodulin dependent kinase II
K_ATP_: ATP-sensitive potassium channels
APD: action potential duration
APA: action potential amplitude
Ca^2+^: calcium
HFD: high fed diet
STZ: streptozotocin
SOD: superoxide dismutase
MDA: malondialdehyde
SGLT2: sodium-glucose co-transporter 2
